# First person – Jennifer Hewitt

**DOI:** 10.1242/dmm.038091

**Published:** 2018-12-04

## Abstract

First Person is a series of interviews with the first authors of a selection of papers published in Disease Models & Mechanisms, helping early-career researchers promote themselves alongside their papers. Jennifer Hewitt is first author on ‘Muscle strength deficiency and mitochondrial dysfunction in a muscular dystrophy model of *Caenorhabditis elegans* and its functional response to drugs’, published in DMM. Jennifer is a PhD student in the lab of Siva Vanapalli at Texas Tech University, Lubbock, USA, investigating using *C. elegans* as a model for studying the mechanisms of and interventions for Duchenne muscular dystrophy.


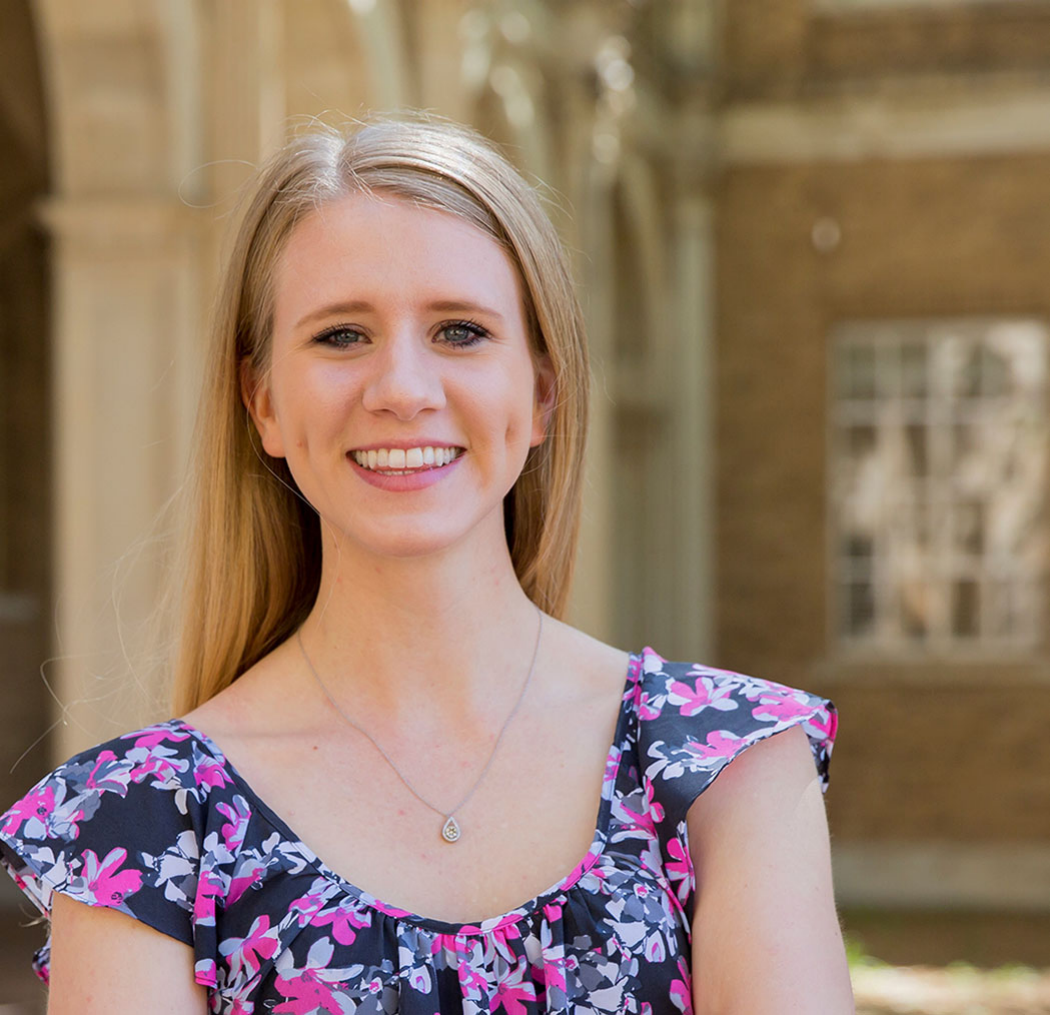


**Jennifer Hewitt**

**How would you explain the main findings of your paper to non-scientific family and friends?**

Duchenne muscular dystrophy (DMD) is a disease in people that results from a mutation that prevents the production of an important protein called dystrophin. This leads to weakness and degradation of muscles. In our study, we surprisingly show that a worm called *C. elegans* also has measurably weak muscle when it has a mutation in dystrophin. We are able to measure *C. elegans* muscle strength using a novel technology called NemaFlex that we developed in our lab. We show that a drug called prednisone, which is used to treat DMD patients, also has beneficial effects on *C. elegans* muscle health. Specifically, prednisone improves muscle strength and energy production.

“[…] the *dys-1(eg33)* mutant of *C. elegans* exhibits muscular weakness as well as mitochondrial dysfunction, deficits that are also seen in patients with DMD.”

**What are the potential implications of these results for your field of research?**

Historically, minimal attention has been given to *C. elegans* as a model for DMD. Most researchers use mouse models of DMD, while some work is done with dog models. However, here we show that *C. elegans* is a relevant model for studying the disease with multiple clinically applicable defects. For the first time, we demonstrate that the *dys-1(eg33)* mutant of *C. elegans* exhibits muscular weakness as well as mitochondrial dysfunction, deficits that are also seen in patients with DMD. The pharmacological response of *C. elegans* to prednisone also shows relevance to the disease, as both muscle strength and abnormal mitochondrial function improve under treatment with prednisone. We think that our results provide a strong argument that researchers should take more interest in using *C. elegans* to study DMD, which may lead to the discovery of new drugs to treat the disease. The DMD research field can also benefit from using *C. elegans* to further study mechanisms of the disease, which has the potential to revolutionize the outlook on treating DMD.

**What are the main advantages and drawbacks of the model system you have used as it relates to the disease you are investigating?**

In general, the advantages of *C. elegans* over other model systems are its short lifespan, low cost of maintenance, quick lifecycle, large brood size and fully sequenced genome, which are useful factors when using this worm to study DMD. *C. elegans* also offers a model system where it is possible to do relatively quick and high-throughput pharmacological screens or genetic studies compared to other model systems. However, *C. elegans* muscle cells cannot regenerate, do not fuse and do not have a typical inflammatory response. While these aspects of *C. elegans* muscle are not entirely negative in the context of studying DMD, this does mean that findings in this worm may not always translate well to humans. However, *C. elegans* is an excellent model for preliminary studies, especially given its clinically relevant defects.

**What has surprised you the most while conducting your research?**

While the strength deficiency of *dys-1* mutants is quite striking, we were perhaps more surprised by the abnormally high use of oxygen in *dys-1(eg33)* mutants and how prednisone returned this to normal. Defects in mitochondrial structure had previously been reported, but no one had reported a functional measure like oxygen use before. There has been much speculation about the mechanism of prednisone in *C. elegans* ever since it was reported that prednisone reduced the number of damaged muscle cells, as described in a 2004 paper by Gaud et al. Prednisone is thought to reduce inflammation in the muscle of DMD-afflicted individuals, but *C. elegans* does not have an inflammation response. Thus, we are able to offer an explanation as to why prednisone improves not only muscle structure, but also function, in DMD-afflicted worms.

**Figure:**
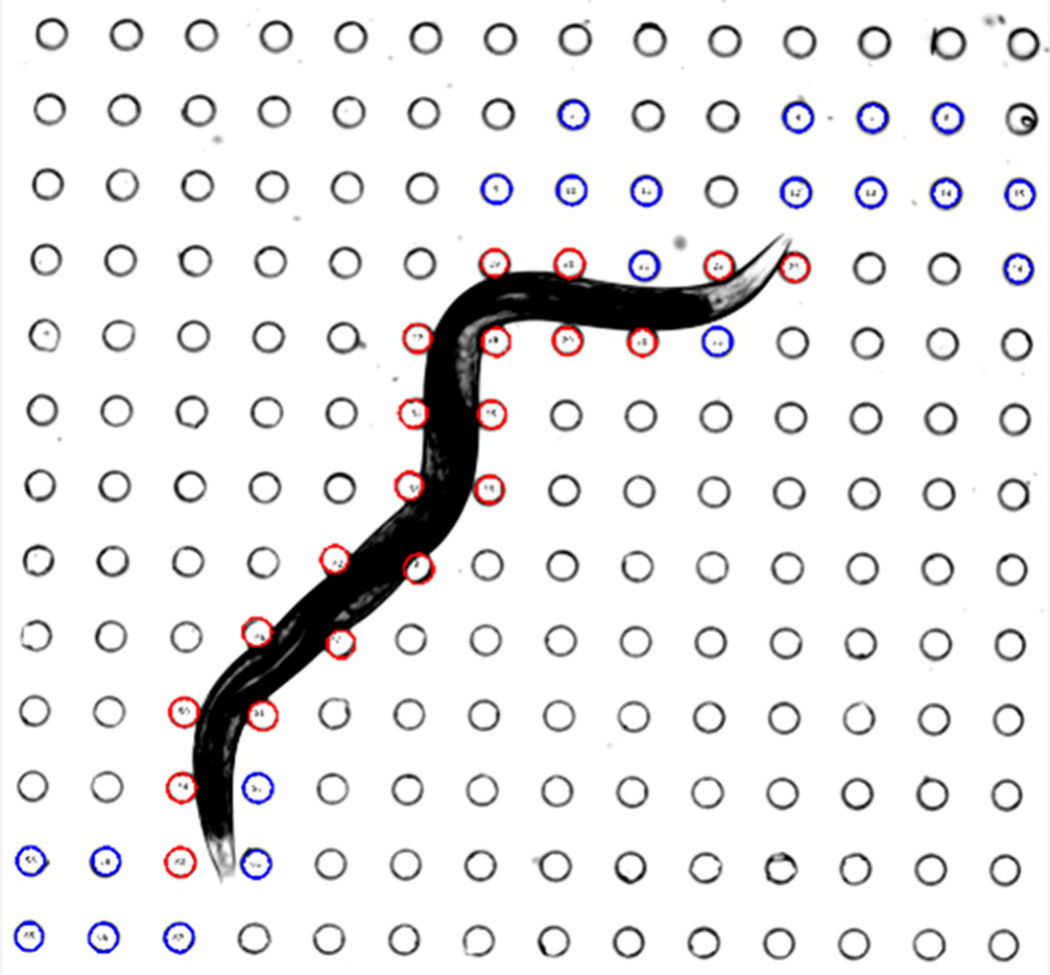
***C. elegans* navigates through an array of pillars inside NemaFlex, our microfluidic device that enables measurement of muscle strength via the deflection of micropillars and a sophisticated image-processing algorithm.**

**Describe what you think is the most significant challenge impacting your research at this time and how will this be addressed over the next 10 years?**

I think the biggest challenge will be convincing people working with different models of DMD to work together to expedite and improve the quality of studies. For example, many people working with mouse or dog models may not be aware of studies like ours that show the value of a worm model of DMD. Similarly, those who work with worm or other invertebrate models should have connections with those working with mouse or dog models, so promising results from worm studies can be translated to other animals. Knowing the advantages and disadvantages of the different systems will allow the best output in the DMD research field. This can be addressed over the next 10 years by the formation of new collaborations, which will hopefully produce several promising new candidate drugs for treating DMD.

“An invested mentor and established collaborators are extremely important for the success of early-career scientists.”

**What changes do you think could improve the professional lives of early-career scientists?**

An invested mentor and established collaborators are extremely important for the success of early-career scientists. Both factors improve the likelihood that young scientists will be able to establish themselves in their research field and obtain funding for their work.

**What's next for you?**

We think that this work is just the beginning of an exciting campaign of research involving *C. elegans* as an improved model for DMD. We would like to form collaborations with academic and private partners to screen novel compounds for treating DMD using *dys-1* mutants and our NemaFlex platform. What is next for me personally is that I will be working to finish my PhD within the next year, after which I hope to continue studying DMD as a postdoctoral researcher or scientist at a private company specializing in understanding and treating muscle diseases.
